# Supplementary material for: Genome-Wide Identification of C2H2 ZFPs and Functional Analysis of BRZAT12 under Low-Temperature Stress in Winter Rapeseed (Brassica rapa)
Source: Int J Mol Sci. 2022 Oct 13;23(20):12218. doi: 10.3390/ijms232012218 (PMC9603636; doi:10.3390/ijms232012218)
Supplement: Supplementary file 1 [file ijms-23-12218-s001.zip › Supplementary Table S1.pdf]

Table S1 Primer sequence information of C2H2 gene family.

Gene ID	Primer sequence 5'-3'	Primer sequence 3'-5'
Bra000245	GTGTACCGCCTTCCACGTCAAC	GTCGGCTCCCTACTCTCTTGGTC
Bra001752	TGTCAAGTACGGTTTCGGAAGAACG	ACTGGGTCGTGATGAAGGCTGAG
Bra002528	GACACATGAGGAGACACAGGAACG	CAACGCTGCCTCCGACAGTAAC
Bra004312	ACTTGCGTCGGTGAACCCATTG	TGATCCAAACGCTGCCGTGAAG
Bra004660	AGTCCTTTAGGTTTGTGGCGTGTAC	GCGGCTCCTTACTCTCCTGGTC
Bra006230	ATGATGGTGGGTGGTGGTGGTG	AGCTGTGGCTCGTCTTCAATGTAG
Bra006691	GTTATTGCCGGAGCCGACGATG	CTCAACCATCCCCAAACTCAGATCC
Bra016900	AACGGAAGTCCTTTGGGTTTGTGG	CGACTCTTTACGCTCCTGCTCATG
Bra020284	CGTTGGTTACAAGAGCGTTATTGCC	ACAAGCCACCCTCTTCCCACTAC
Bra033724	TTGCGTCTGCGATGGATGAACTAC	CCTCCTCCACCACCTTCTCCTC
β-actin	TGTGCCAATCTACGAGGGTTT	TTTCCCGCTCGGCTGTTGT
