# Supplementary figures and images for: Genome-Wide Identification of C2H2 ZFPs and Functional Analysis of BRZAT12 under Low-Temperature Stress in Winter Rapeseed (Brassica rapa)
Source: Int J Mol Sci. 2022 Oct 13;23(20):12218. doi: 10.3390/ijms232012218 (PMC9603636; doi:10.3390/ijms232012218)

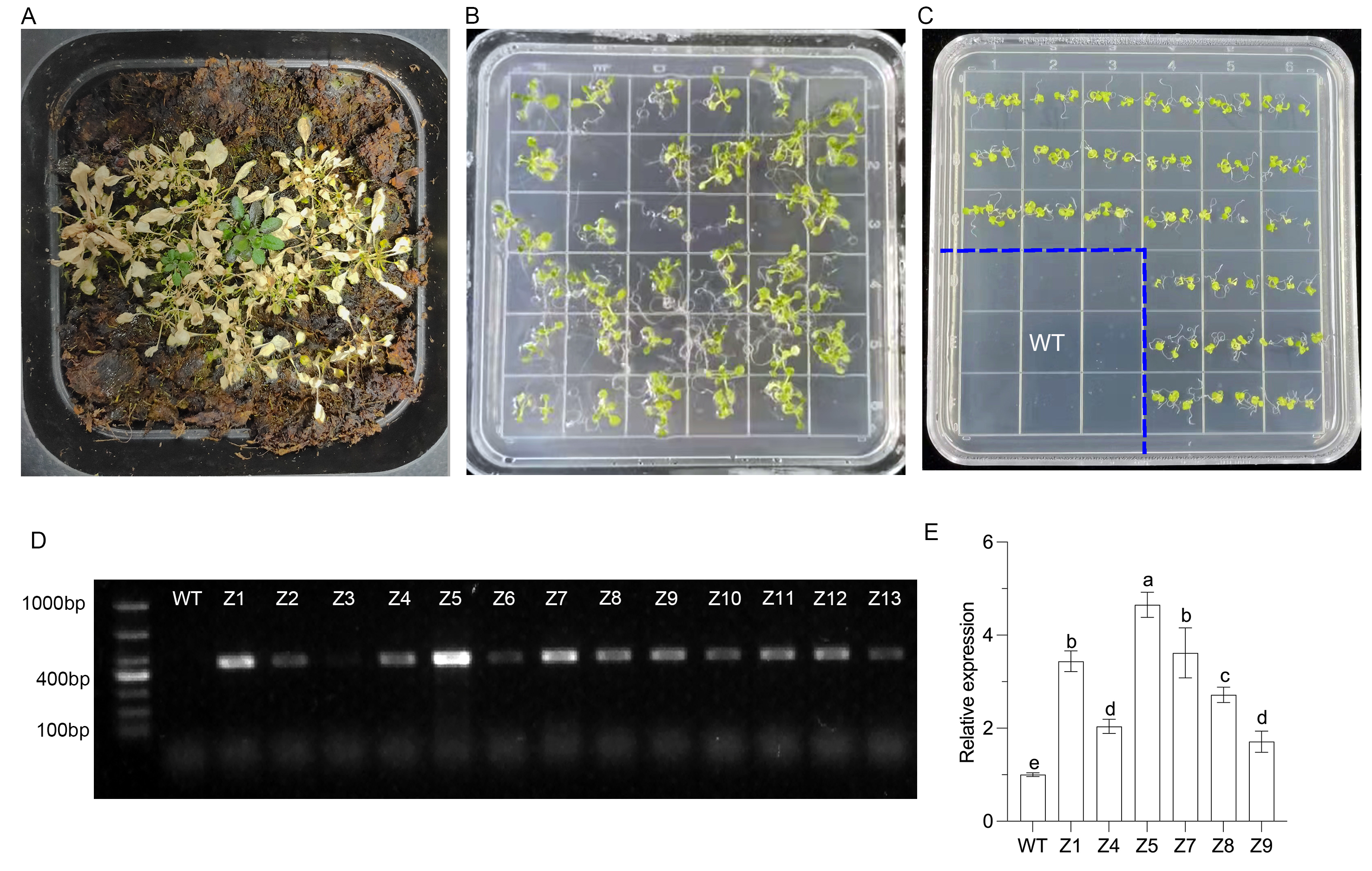

Supplement: Supplementary file 1 [file ijms-23-12218-s001.zip › Supplementary Figure S1.jpg]
